# CT texture analysis predicts abdominal aortic aneurysm post-endovascular aortic aneurysm repair progression

**DOI:** 10.1038/s41598-020-69226-1

**Published:** 2020-07-23

**Authors:** Ning Ding, Yunxiu Hao, Zhiwei Wang, Xiao Xuan, Lingyan Kong, Huadan Xue, Zhengyu Jin

**Affiliations:** 1 Department of Radiology, Peking Union Medical College Hospital, Peking Union Medical College, Peking Union Medical College and Chinese Academy of Medical Sciences, Shuai Fu Yuan 1#, Dongcheng Dist, Beijing, 100730 People’s Republic of China; 2 Neusoft Medical Systems Co. Ltd, Beijing, People’s Republic of China

**Keywords:** Diseases, Cardiovascular diseases, Vascular diseases, Aneurysm

## Abstract

The aim of this study is to investigate the role of early postoperative CT texture analysis in aneurysm progression. Ninety-nine patients who had undergone post-endovascular aneurysm repair (EVAR) infra-renal abdominal aortic aneurysm CT serial scans were enrolled from July 2014 to December 2019. The clinical and traditional imaging features were obtained. Aneurysm texture analysis was performed using three methods—the grey-level co-occurrence matrix (GLCM), the grey-level run length matrix (GLRLM), and the grey-level difference method (GLDM). A multilayer perceptron neural network was applied as a classifier, and receiver operating characteristic (ROC) curve analysis and area under the curve (AUC) analysis were employed to illustrate the classification performance. No difference was found in the morphological and clinical features between the expansion (+) and (−) groups. GLCM yielded the best performance with an accuracy of 85.17% and an AUC of 0.90, followed by GLRLM with an accuracy of 87.23% and an AUC of 0.8615, and GLDM with an accuracy of 86.09% and an AUC of 0.8313. All three texture analyses showed superior predictive ability over clinical risk factors (accuracy: 69.41%; AUC: 0.6649), conventional imaging features (accuracy: 69.02%; AUC: 0.6747), and combined (accuracy: 75.29%; AUC: 0.7249). Early post-EVAR arterial phase-derived aneurysm texture analysis is a better predictor of later aneurysm expansion than clinical factors and traditional imaging evaluation combined.

## Introduction

Abdominal aortic aneurysm (AAA) is a prevalent irreversible cardiovascular disease with a high mortality rate that needs immediate surgical intervention. Endovascular aortic aneurysm repair (EVAR) is the preferable choice for patients with AAA as a minimally invasive procedure, but intensive surveillance is recommended to detect possible postoperative aneurysm sac enlargement, the most recognised indicator of AAA rupture^1^ , and for which secondary intervention is often performed to prevent deadly progression^2,3^.

Computed tomography (CT) is commonly used after EVAR for follow-up surveillance, and enhanced CT is usually performed as the first postoperative imaging modality to evaluate the outcome of EVAR surgery and make further individual treatment plans^1,4^. Endoleak can easily be observed on postoperative CT and is detected in nearly 32% of AAAs^5^. A classic sign of an endoleak is the observation of contrast agent overflow out of the stent-graft.

Nevertheless, the long-term significance of the first noticed endoleak is debatable, transient endoleaks can be resolved spontaneously without medical intervention^6,7^. Additionally, late-onset endoleaks are typically detected 6 months later after EVAR and are usually occult on the first CT scan^8,9^. Thus, the detection of endoleaks on the first operative CT alone is limited to guide clinical decisions, and periodic imaging follow-up is still needed to monitor the evolution of AAA^10^, thus raising the medical expenditure, ionisation radiation, and potential harm to renal function.

CT texture analysis is an emerging imaging post-processing technique that can provide textural features by quantifying tissue grey-level patterns. Compared with conventional imaging alone, adding CT texture analysis can enhance the diagnosis ability in benign and malignant neoplasm differentiation^11^, the grading of tumours^12,13^ and the prediction of a therapeutic response^14,15^. However, to our knowledge, no study has performed in vivo CT texture analysis to test the predictive value of the first postoperative CT for future aneurysm expansion. The current study was aimed to determine whether CT texture analysis plays a role in the first postoperative CT to predict aneurysm progression.

## Methods

### Ethical consideration

This study was approved by the institutional review board of Peking Union Medical College Hospital. The requirement of informed consent from patients was waived by the institutional review board. All the procedures were performed following the ethical standards of the institutional and/or national research committee and with the 1964 Helsinki declaration and its later amendments or comparable ethical standards.

### Patients

**Figure 1:**
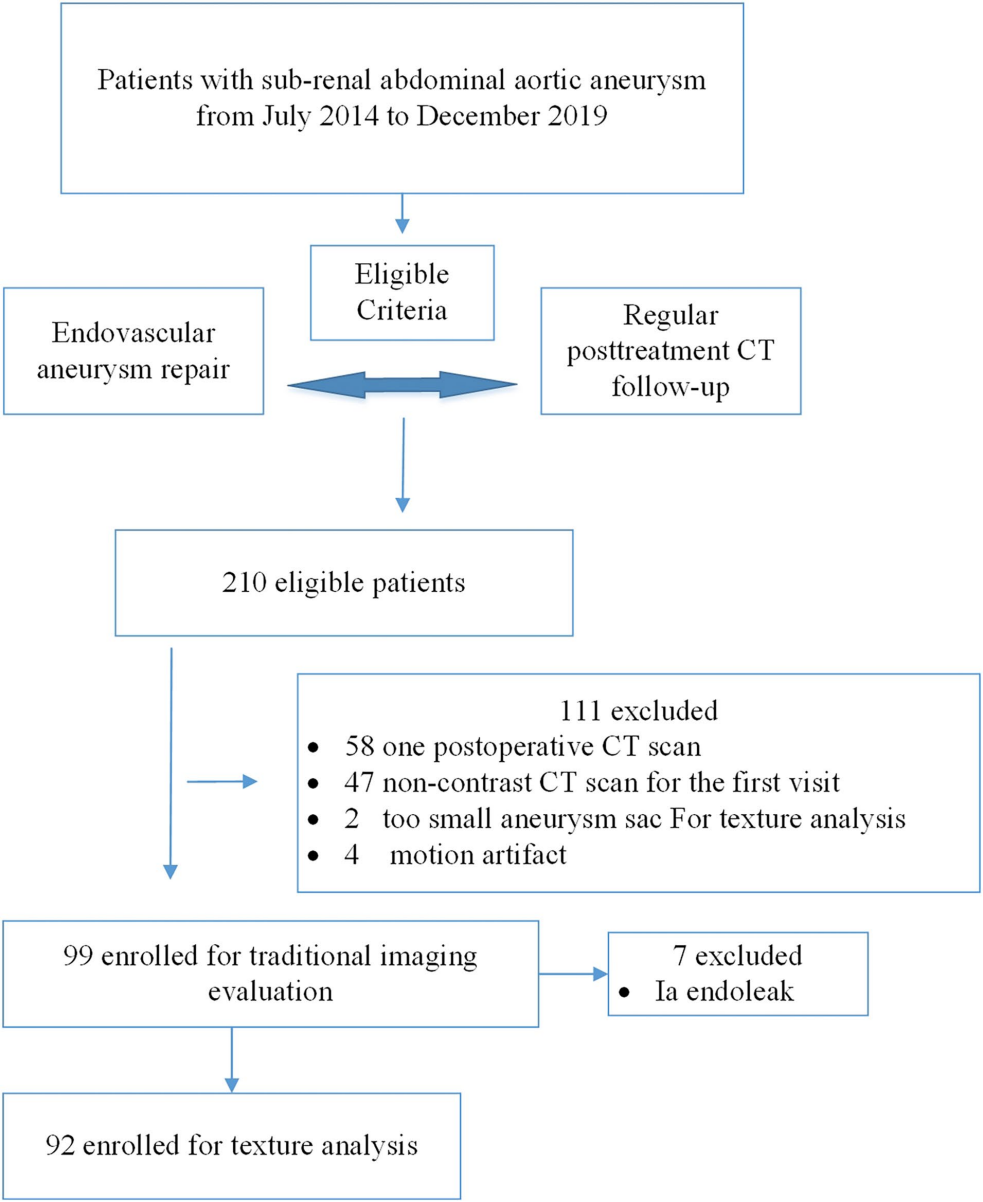
Flowchart of the current study.

This was a retrospective study. The inclusion criteria were as follows: (1) patients with infra-renal abdominal aortic aneurysm; (2) patients who had undergone intra-abdominal aortic aneurysm repair; (3) at least two regular postoperative (approximately 3 and 12 months) follow-up CT examination of the abdominal and pelvis; (4) the first CT scan was contrast enhanced (Fig. 1). From July 2014 to December 2019, 210 patients who had undergone post-EVAR infra-renal abdominal aortic aneurysm CT scans were identified by searching the electronic medical records (EMR) system and picture archiving and communication system. Fifty-eight were excluded because they did not undergo postoperative CT scans twice, 47 were excluded because they had only undergone non-contrast CT scan for the first scan, two were excluded because the aneurysm sac area was too small for effective texture analysis, four were excluded because of severe motion artefacts that could influence texture analysis. Finally, 99 patients were included in the analysis.

### Clinical parameters and conventional imaging characteristics

The clinical risk factors associated with aneurysm rupture were recorded before the EVAR procedure, including age, gender, preoperative blood pressure, hypertension history and duration, heart disease history, diabetes history, smoking history and time of duration, current smoking status, alcohol consumption history and current drinking status, total cholesterol level, triglyceride level, high-density lipoprotein cholesterol level, and low-density lipoprotein cholesterol level. The conventional imaging characteristics currently applied in daily aneurysm evaluation were evaluated by two radiologists with 10 and 6 years of vasculature reading experience. The conventional imaging features were comprised of the first-interval maximum aneurysm diameter, the aneurysm volume measured and whether the post-surgery aneurysm sac endo-leak exists, and, if so, its subtype. The aneurysm volume was evaluated by the three dimensional (3D) post-processing tool named Advanced Vessel Analysis (AVA, Philips Healthcare, Netherlands) software on a dedicated workstation (IntelliSpace Portal Version 9.0.4, Philips Healthcare, Netherlands).

### CT image acquisition

All abdominal aortic contrast-enhanced CT scans were performed using a first-generation dual-source CT (SOMATOM Definition, Siemens Healthcare, Germany). The institutional protocol was biphasic, a head-first supine position, and a scanning range from the diaphragm level to the pubic symphysis level. The contrast medium iopromide (370 mgI/ml; Shanghai Bolaike Xinyi Pharmaceutical Co., Ltd.) was injected using a high-pressure syringe through the median right elbow at a rate of 4.0 ml/s.

A bolus tracking technique was used with a triggering threshold of 100 Hu; the trigger level was the abdominal aorta. Other protocol parameters were as follows: tube voltage, 120 kV; tube current, 200 mAs; rack rotation time, 330 ms; collimation, 2 × 32 × 0.6 mm; pitch, 0.8 mm.

### Image segmentation and feature extraction

**Figure 2:**
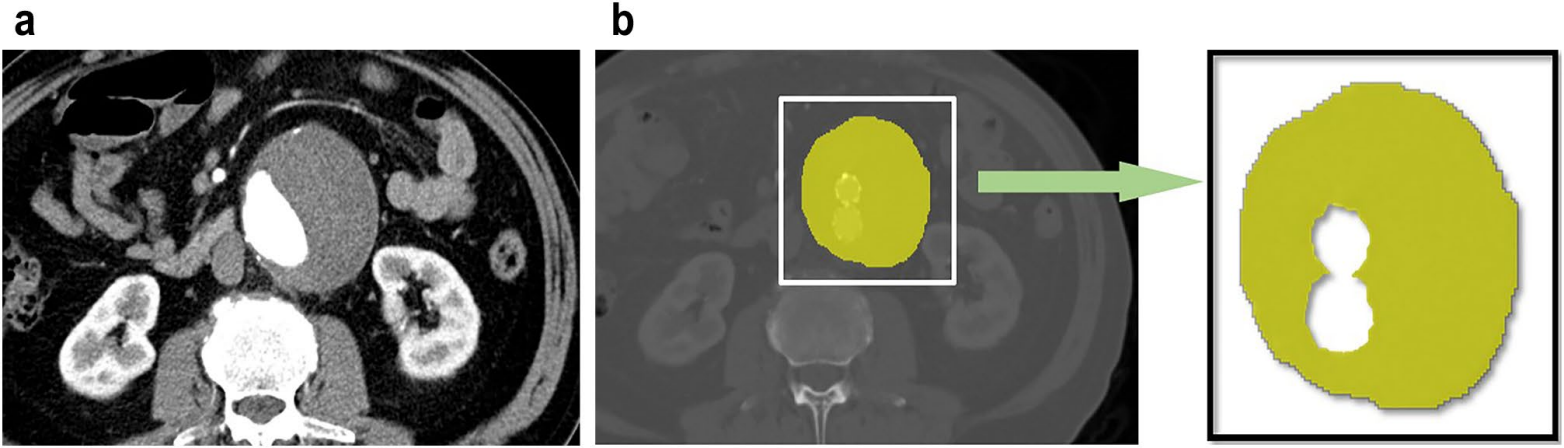
Segmentation and feature extraction process. (**a**) Maximal axial plane of preoperative abdominal aortic aneurysm in contrast-enhanced CT angiography. (**b**) Maximal axial plane of post EVAR abdominal aortic aneurysm in contrast-enhanced CT angiography. Two radiologists reviewed all the axial contrast CT scans and defined the maximum aneurysm cross section using MATLAB (R2016a, Mathworks, United States) software to manually outline the aneurysm sac area outside the stent. Abbreviation: EVAR, endovascular aneurysm repair.

The largest axial plane of the aneurysm sac was selected on the CT images of the arterial phase, and MATLAB software (R2016a, Mathworks, United States) was used to manually outline the largest axial section of the aneurysm outside the stent (Fig. 2). Next, the grey-level matrix was used to extract the texture features of the thrombus-defined region of interest (ROI), and then the texture features were input into the classifier generated by the neural network to obtain the final classification results.

In this paper, we adopted three grey-level matrices to extract texture features: the grey-level co-occurrence matrix (GLCM), the grey-level run length matrix (GLRLM) and the grey-level difference method (GLDM) (Table 1). These three matrices can obtain second-order or higher order statistical relationships of grey values between the pixel pairs or groups, and then describe the texture characteristics of the CT image.

**Table 1:** Detailed features in three greyscale matrices.

GLCM matrix	GLDM matrix	GLRLM matrix
Energy	Contrast	Short-run emphasis
Correlation	Angular second moment	Long-run emphasis
Inertia	Entropy	Grey-level nonuniformity
Entropy	Mean	Run percentage
Inverse difference moment	Inverse difference moment	Run length nonuniformity
Sum average		Low-grey-level run emphasis
Sum variance		High-grey-level run emphasis
Sum entropy		
Difference average		
Difference variance		
Difference entropy		
Two information measures of correlation		

Grey-Level Co-occurrence Matrix = GLCM.

Grey-Level Run Length Matrix = GLRLM.

Grey-Level Difference Method = GLDM.

#### GLCM

The GLCM is an estimation of the second-order joint conditional probability-density function f((i,j)⁄(d,θ)). This function characterises the spatial interrelationships of the grey values in an image. The values of the co-occurrence matrix elements represent the probability of changing from grey level i to grey level j given that they are separated by distance d and the direction is given by angle θ (usually θ = 0°, 45°, 90°, and 135°).

GLCM describe the distances and angles of the pixels. In the current application, the aneurysm capsule is assumed to have the characteristics of an isotropic texture distribution. Thus, we calculate the average distribution of eigenvalues in four corners; at the same time, to reduce the impact of random noise, the grey level is reduced to 16 before calculating the feature matrix. A set of features is extracted using the GLCM matrix and comprises 13 types: energy, correlation, contrast, entropy, deficit moment, sum average, sum variance, sum entropy, difference average, difference variance, difference entropy and two information measures.

#### GLRLM

The GLRLM extracts higher order statistical texture information. For a given image, a run length matrix is a two-dimensional matrix in which each element p((i,j)⁄θ) represents the total number of runs with pixels of grey value i and run length j in a certain direction θ.

The following seven features are calculated: short run advantage, long run advantage, gray level nonuniformity, run percentage, long run nonuniformity, low gray level run advantage and high gray level run advantage.

#### GLDM

The GLDM obtains first-order statistics of local property values. The GLDM is based on the occurrence of two pixels with a given absolute difference in the grey level and which are separated by a specific displacement δ.

GLDM is based on the probability of the existence of two pixels with a special relationship, referring to the fixed displacement difference and grey-value difference between the two pixels. This matrix extracts five features: correlation, angular second-order moment, entropy, mean value and inverse moment. Similar to the GLCM method, the assumption of isotropic texture distribution in the aneurysm capsule is considered, and the average distribution in four directions is calculated.

### Reference standard

**Figure 3:**
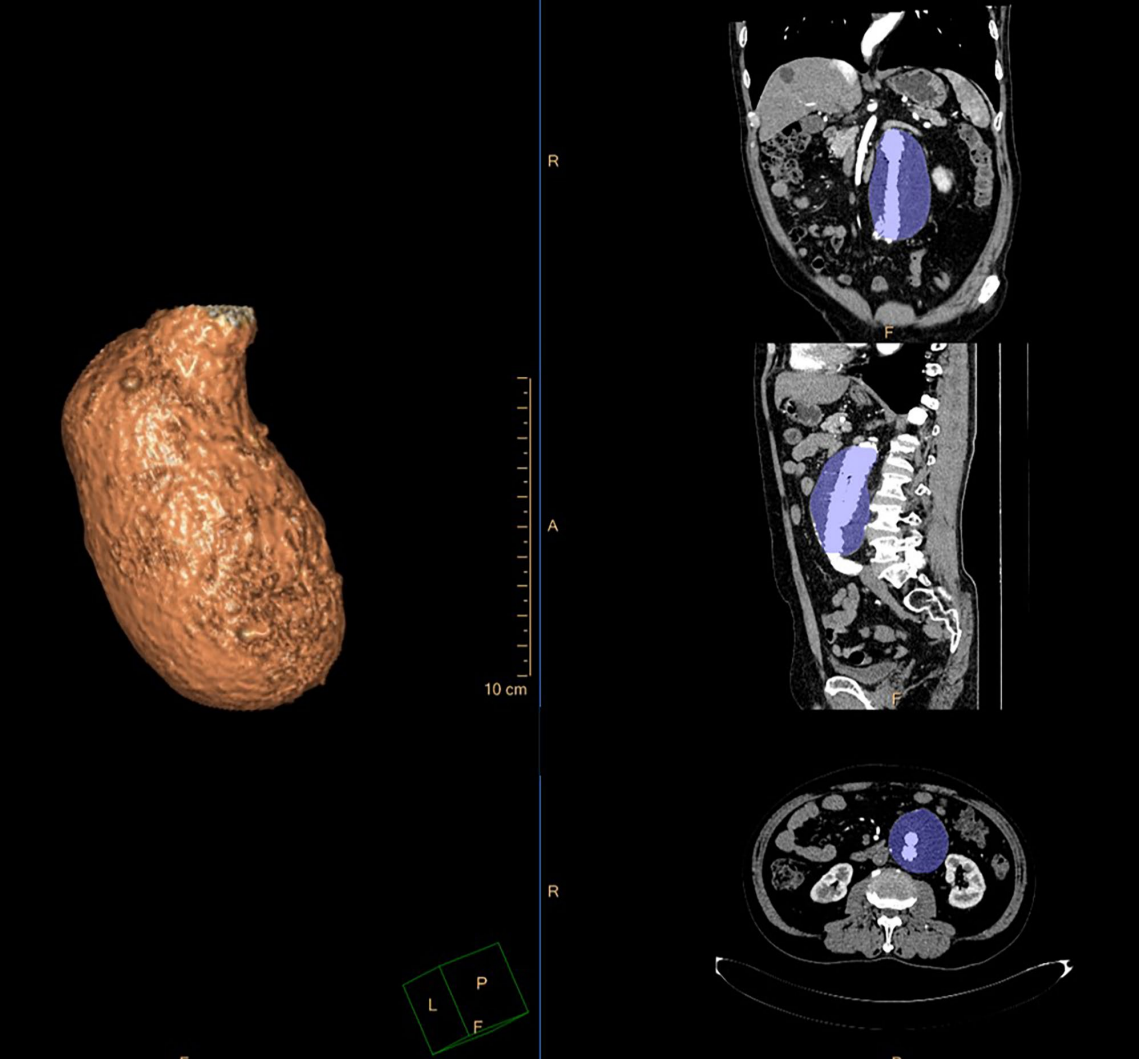
Aneurysm volume measurement by the three dimensional (3D) post-processing software (IntelliSpace Portal Version 9.0.4, Philips Healthcare, Netherlands). (**a**) Visual reconstruction technique. (**b**–**d**) Multi-planar reconstruction. (**b**) Coronal plane. (**c**) Sagittal plane. (**d**) Axial plane. The aneurysm boundary was manually delineated along the outer contour using three-dimensional multi-planar reconstruction.

Aneurysm volume expansion was used as the reference standard, and it is derived from the volume change between the first and second postoperative CT scans. The enhanced arterial phase images were imported into 3D post-processing software (Philips IntelliSpace Portal, Royal Philips, Amsterdam, Netherlands) to measure the aneurysm volume (Fig. 3). The aneurysm boundary was manually delineated along the outer contour, ranging from the level of the renal artery on the lower side to the level of the total bifurcation on both sides. All calcifications and mural thrombi within the aneurysm sac were included; however, any branch vessels derived from the aneurysm was excluded.

The volume of the aneurysm measured at the first postoperative follow up was recorded as V1, and the volume of the aneurysm measured at the second postoperative follow up was recorded as V2. If the (V2-V1)/V1 is greater than 2%, it was recorded as an aneurysm expansion^16–18^; otherwise, it was recorded as a stable or shrunken aneurysm.

### Statistical analysis

The purpose of the current study was to explore the potential role of the texture analysis of early CT in the prediction of aneurysm expansion.

In testing the significance of clinical risk factors and conventional imaging parameters, the one-sample Kolmogorov–Smirnov test was used for normality test, and independent samples T test was applied for ordinal variables. SPSS (version 22.0; SPSS Inc, IBM Co, Chicago, Illinois) was applied. A *P* value < 0.05 was deemed to indicate a statistically significant difference. All *P* values were two-sided.

The study cohort was divided as a ratio of 70:15:15 into training, validation and test groups using MatLab v. R2015a (MathWorks, Natick, Massachusetts). This ratio is necessary to construct an effective classifier. Cross validation is a common method to expand datasets when the amount of data is relatively small. To improve the evaluation of the performance of the feature sets, the cross validation was repeated 100 times averaging the results.

The classifier used in this study was a three-layer backpropagation neural network^19^. The neural network mapped traditional imaging, clinical factors and three texture features (GLCM, GLDM, and GLRLM) into values through functions, and then drew the ROC by modifying the classification threshold of this feature.

## Results

Ninety-nine patients were included in our cohort, with 87 males and 12 females aged 68 ± 8 years (age range, 46–89 years). Early abdominal aortic CTA was performed 3 months (range, 2.3 to 3.6 months) after EVAR, and the first-interval AAA volume was 156.9 ± 133.6 cc. After an average interval of one year (range, 11.6 to 14.1 months), the second-interval AAA volume was 158.6 ± 162.9 cc. Among all the patients, 38 had aneurysm expansion and 61 had a shrunken or stable aneurysm, they were refered as expansion (+) group and expansion (−) group respectively. The relative volume change in the expansion group was 9.8% ± 9.9% and that in the non-expansion group was − 8.7% ± 10.7%. Overall, 19 visualised endoleaks were recorded in the first-interval CTAs, among which 7 were type I and 12 were type II endoleaks. Twelve endoleaks were observed in the expansion (+) group, and seven endoleaks were diagnosed in the expansion (−) group.

Interobserver agreement between the two readers was excellent for the evaluation of the early maximal AAA diameter (mean, 5.1 ± 1.5 cm vs. 5.0 ± 1.4 cm; *P* = 0.119; ICC = 0.956), CT-reported endoleak (*P* = 0.41; ICC = 0.932), first-interval aneurysm volume (mean, 156.9 ± 133.6 cc vs. 160.5 ± 132.9 cc; *P* = 0.108; ICC = 0.986) and second-interval aneurysm volume (mean, 158.6 ± 162.9 cc vs. 166.6 ± 182.9 cc; *P* = 0.008; ICC = 0.985), and all values showed good reproducibility. The results from reader one were used for further analysis.

Binary logistic regression was carried out using conventional imaging features and clinical aneurysm risk factors, and the variable selection criteria were based on clinical importance and P < 0.1 in univariable analysis (Tables 2, 3). The endoleak, maximal diameter and alcohol assumption history were input for further logistic regression. Among all 99 aneurysms, neither the traditional imaging parameters (Table 2) nor the clinical risk factors (Table 3) showed a statistical significance between the expansion (+) and (−) groups. The prediction probability was generated and used in ROC curve analysis; all traditional imaging parameters attained an AUC of 0.6747, clinical risk factors attained an AUC of 0.6488, and the combination model demonstrated an AUC of 0.7249.

**Table 2:** Conventional CT features in the study cohort.

	All N = 99	Aneurysm expansion (+) group n = 38	Aneurysm expansion (−) group n = 61	Univariable analysis sig	OR	Logistic regression sig
First postoperative maximal aneurysm diameter (mm)	50.9 ± 14.8	52.7 ± 17.3	49.7 ± 13.0	0.337	0.998	0.917
CT-reported endoleak	No endoleak: 80	No endoleak: 26	No endoleak: 54	0.063	1.0	0.071
	Type I endoleak: 7	Type I endoleak: 6	Type I endoleak: 1		11.514	
	Type II endoleak: 12	Type II endoleak: 6	Type II endoleak: 6		2.116	
First-interval aneurysm volume (cc)	156.9 ± 133.6	167.1 ± 168.7	150.6 ± 107.3	0.553	…	…
Second-interval aneurysm volume, (cc)	158.6 ± 162.9	191.8 ± 225.2	137.9 ± 104.6	0.110	…	…

**Table 3:** Clinical risk factors for AAA in the study cohort.

	All N = 99	Aneurysm expansion (+) group n = 38	Aneurysm expansion (−) group n = 61	Univariable analysis sig	OR	Logistic regression sig
**Demographic factors for AAA**						
Gender	M: 87	M: 33	M: 54	0.805	…	…
	F: 12	F: 5	F: 7			
Age (years)	68.5 ± 8.3	70.0 ± 7.4	67.5 ± 8.8	0.159	…	…
**Cardiovascular-related status**						
Hypertension	No: 36	No: 12	No: 24	0.519	…	…
	Yes: 60	Yes: 24	Yes: 36			
	NA: 3	NA: 2	NA: 1			
Hypertension duration (years)	9.7 ± 13.7	11.2 ± 12.2	8.7 ± 14.6	0.389	…	…
Systolic pressure (mmHg)	146.3 ± 26.4	146.9 ± 25.5	145.9 ± 27.2	0.858	…	…
Diastolic pressure (mmHg)	83.6 ± 16.1	82.3 ± 16.9	84.0 ± 15.6	0.763	…	…
Heart disease	No: 66	No: 22	No: 44	0.147	…	…
	Yes: 33	Yes: 16	Yes: 17			
**Metabolism-related index**						
Diabetes	No: 84	No: 30	No: 54	0.130	…	…
	Yes: 14	Yes: 8	Yes: 6			
	NA: 1		NA:1			
Smoking history	No: 37	No: 15	No: 22	0.783	…	…
	Yes: 61	Yes: 23	Yes: 38			
	NA: 1		NA: 1			
Current Smoking Status	No: 64	No: 23	No: 41	0.186	…	…
	Yes: 31	Yes: 13	Yes: 19			
	NA: 3	NA: 2	NA: 1			
Smoking duration (years)	21.6 ± 20.4	21.5 ± 20.5	21.6 ± 20.5	0.982	…	…
Alcohol consumption	No: 66	No: 21	No: 45	0.062	2.014	0.132
	Yes: 31	Yes: 16	Yes: 15			
	NA: 2	NA: 1	NA: 1			
Current consumption status	No: 78	No: 28	No: 50	0.361	…	…
	Yes: 19	Yes: 9	Yes: 10			
	NA: 2	NA: 1	NA: 1			
Total cholesterol (mmol/L)	4.2 ± 1.0	4.3 ± 1.3	4.2 ± 0.9	0.921	…	…
Triglyceride (mmol/L)	1.6 ± 0.9	1.6 ± 1.0	1.6 ± 0.9	0.916	…	…
High-density lipoprotein cholesterol (mmol/L)	1.0 ± 0.3	1.0 ± 0.4	0.9 ± 0.3	0.168	…	…
Low-density lipoprotein cholesterol (mmol/L)	2.5 ± 1.0	2.6 ± 1.2	2.5 ± 0.7	0.674	…	…

The type 1a endoleak is a well established predictor for aneurysm expansion^5^, and in many cases re-intervention are needed to repair the type 1a endoleak^20^. The odd ratio for expansion of the aneurysm with a type 1a endoleak is 11.5, which was much larger than any other factors, therefore we excluded all seven type 1a endoleak in the following texture analysis. Totally ninety-two patients were included for texture analysis, with thirty-two in expansion group and sixty in non-expansion group.

**Figure 4:**
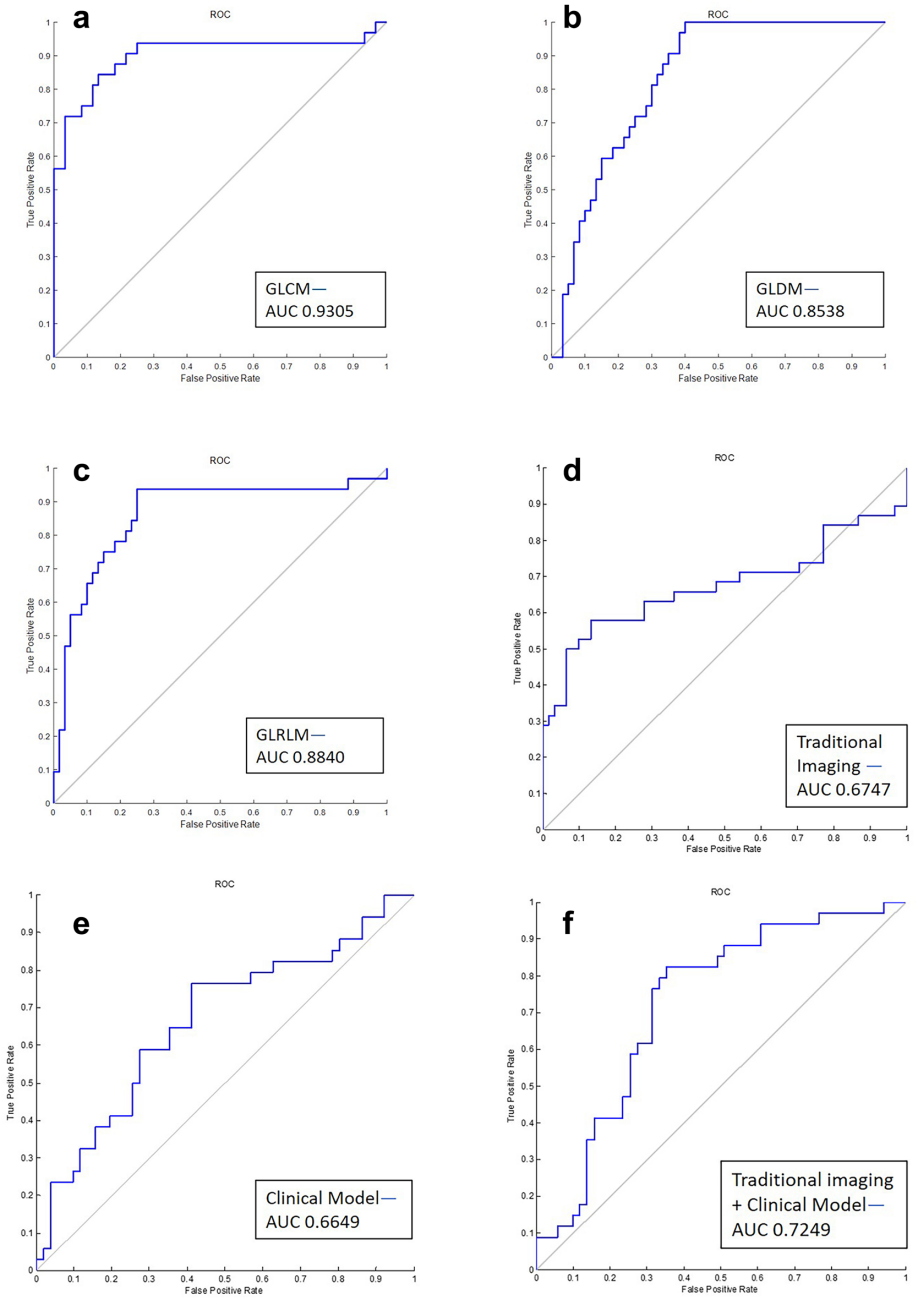
ROC curves among the different models. (**a**) The GLCM matrix attained the highest AUC of 0.90, with a sensitivity of 0.8438 and a specificity of 0.8559. (**b**) The GLDM matrix attained AUC of 0.8313, with a sensitivity of 1 and a specificity of 0.7867. (**d**) The GLRLM matrix attained AUC of 0.8615, with a sensitivity of 0.9375 and a specificity of 0.8375. (**d**) Traditional imaging evaluation attained the highest AUC of 0.6747, with a sensitivity of 0.5789 and a specificity of 0.8689. (**e**) The clinical risk models attained the highest AUC of 0.6649, with a sensitivity of 0.7647 and a specificity of 0.5882. (**f**) The traditional imaging and clinical risk factors combined attained the highest AUC of 0.7249, with a sensitivity of 0.8235 and a specificity of 0.6471. *AUC* area under the curve.

The GLCM texture feature yielded a performance with a discrimination accuracy of 85.17% and an AUC of 0.90, the GLDM feature attained an accuracy of 86.09% and an AUC of 0.8313, and the GLRLM feature attained an accuracy of 87.23% and an AUC of 0.8615. The results of the optimal cutoff values for different texture features, the clinical and traditional imaging models obtained from ROC curves and the sensitivity/specificity with area under the receiver operating characteristic curve to predict aneurysm expansion are shown in Table 4 and Fig. 4.

**Table 4:** Optimal cutoff values from ROC curves and sensitivity/specificity to predict aneurysm expansion.

Texture features	AUC	Cutoff values	Sensitivity	PPV	NPV	Specificity	Accuracy
GLCM	0.9010	0.3950	0.8438	0.7575	0.9113	0.8559	0.8517
GLDM	0.8313	0.2610	1	0.7143	1	0.7867	0.8609
GLRLM	0.8615	0.3371	0.9375	0.7547	0.9617	0.8375	0.8723
Conventional imaging model	0.6747	0.4014	0.5789	0.8764	0.5625	0.8689	0.6902
Clinical model	0.6649	0.0174	0.7647	0.7358	0.6250	0.5882	0.6941
Conventional imaging model + clinical model	0.7249	0.4598	0.8235	0.7778	0.7097	0.6471	0.7529

## Discussion

We performed CT texture analysis in this study to quantitatively reflect the homogeneity of CT values of the aneurysm sac outside the stent. The expansion of the aneurysm body is related to endoleak and its augmented internal pressure, which could manifest as an uneven distribution of the contrast agent and poor CT value uniformity. By contrast, the pressure in the stable aneurysm sac is low, with less or no contrast medium outflow; thus, the uniformity of the CT values is good.

Our findings demonstrated that CT texture analysis has an excellent performance in predicting post EVAR AAA expansion. Texture analysis showed a much better performance to identify this uniformity than radiologists’ experiences.

All three texture matrices showed preferable risk stratification capability (AUC, 0.83–0.90) over traditional imaging parameters (AUC, 0.69), clinical risk factors (AUC, 0.65) and even the combination (AUC, 0.72).

Contrast-enhanced CT after EVAR is the most commonly used follow-up imaging modality to evaluate endoleak and other postoperative complications. Missing the feasible treatment window period could lead to aneurysm rupture and other serious consequences, and overestimating the AAA progression risk could add to unnecessary ionisation radiation, cost and psychological burden^1,4^. To this end, our study established a promising approach to identify high-risk AAA that needs secondary intervention in the early postoperative stage. Our results imply that texture analysis could serve as a tool for AAA expansion risk prediction and a bridge for an individualised treatment plan.

The increased aneurysm volume is directly related to the risk of aneurysm expansion^21,22^. In this study, the change of aneurysm volume was used as the reference standard. Compared with the application of the change in the maximum diameter of the aneurysm, the aneurysm volume can be a more sensitive predictor for aneurysm change and the most significant indicator for intervention^18,23^ The cutoff value of the relative volume change to classify any aneurysm as expansion or non-expansion was set at 2%. As stated in the previous literature, a relative volume change larger than 2% after EVAR may require re-intervention, while a smaller than 2% change is considered relatively stable and requires no surgical intervention^24,25^; additionally, the average variability is less than 2% for well-trained evaluators^26,27^.

Texture analysis is a promising method in risk prediction and has been applied in various medical fields^28–30^, however, only a few studies have been carried out on the prediction value of AAA progression. Carl W. Kotze and colleagues applied CT heterogeneity analysis in fifty small aortic aneurysms without surgical intervention and identified the predictive role of texture analysis for aneurysms at risk of expansion^31^. G. García’s team evaluated four texture analysis methods and three classification schemes in three post EVAR patients and proposed a computer-aided system based on texture analysis^19^. Compared with previous texture analysis studies, our research demonstrated the added value of texture analysis in post EVAR evaluation prediction using a larger cohort size.

In general clinical settings, the endoleak^5,32^ and maximal aneurysm diameters^1, 4^ are considered the most significant risk features for rupture prediction. Population risk factor analysis of AAA showed that AAA mortality is related to systolic blood pressure, cholesterol, and smoking prevalence with statistical significance^33,34^.

In the current study, we incorporated traditional imaging features and clinical factors into the prediction model and found that the predictive model using texture parameters demonstrated superior predictive power than the model using both conventional imaging features and clinical factors.

Our study had several limitations. First, at our institution, abdominal aortic CTA was performed on the same CT scanner; thus, further study is needed to evaluate the results using other scanners. Second, the ROIs were drawn on a single maximum axial slice CT, not on the whole aneurysm; although the texture features of the whole aneurysm could be more informative, 2D images are more practical in clinical settings. Third, the patients with endoleak were all classified as type I and type II because these two types are the most common forms. Furthermore, this was a single-centre retrospective study; multi-centre prospective studies are needed to further confirm the performance of texture analysis.

## Conclusion

Texture analysis of early postoperative CT was shown to be a better predictor for later aneurysm growth than traditional clinical and imaging evaluation. The proposed method has a potential role to assist physicians in making individualised follow-up plans and intervention decisions.
